# Nanodroplet-Based Reagent Delivery into Water-in-Fluorinated-Oil Droplets

**DOI:** 10.3390/bios13080768

**Published:** 2023-07-28

**Authors:** Bo Zhu, Zhe Du, Yancen Dai, Tetsuya Kitaguchi, Sebastian Behrens, Burckhard Seelig

**Affiliations:** 1 Department of Biochemistry, Molecular Biology and Biophysics, University of Minnesota, Minneapolis, MN 55455, USA; 2 BioTechnology Institute, University of Minnesota, St. Paul, MN 55108, USA; 3 Laboratory for Chemistry and Life Science, Institute of Innovative Research, Tokyo Institute of Technology, Yokohama 226-8503, Japan; 4 Center for Environmental Health Risk Assessment and Research, Chinese Research Academy of Environmental Sciences, Beijing 100012, China; 5 Department of Civil, Environmental, and Geo-Engineering, University of Minnesota, Minneapolis, MN 55455, USA

**Keywords:** reagent delivery, nanodroplet, microdroplet, biosensor, microfluidic device

## Abstract

In vitro compartmentalization (IVC) is a technique for generating water-in-oil microdroplets to establish the genotype (DNA information)–phenotype (biomolecule function) linkage required by many biological applications. Recently, fluorinated oils have become more widely used for making microdroplets due to their better biocompatibility. However, it is difficult to perform multi-step reactions requiring the addition of reagents in water-in-fluorinated-oil microdroplets. On-chip droplet manipulation is usually used for such purposes, but it may encounter some technical issues such as low throughput or time delay of reagent delivery into different microdroplets. Hence, to overcome the above issues, we demonstrated a nanodroplet-based approach for the delivery of copper ions and middle-sized peptide molecules (human p53 peptide, 2 kDa). We confirmed the ion delivery by microscopic inspection of crystal formation inside the microdroplet, and confirmed the peptide delivery using a fluorescent immunosensor. We believe that this nanodroplet-based delivery method is a promising approach to achieving precise control for a broad range of fluorocarbon IVC-based biological applications, including molecular evolution, cell factory engineering, digital nucleic acid detection, or drug screening.

## 1. Introduction

In vitro compartmentalization (IVC) was first developed by Dan Tawfik and Andrew Griffiths in 1998 for high-throughput directed evolution [1]. In this approach, a genotype–phenotype linkage is achieved due to a physical barrier provided by artificial reaction compartments such as water-in-oil droplets, which mimic the cells of living organisms. IVC-based methods have already been used to perform various biological applications including evolving enzymes [1,2,3,4,5,6,7,8], prototyping genetic circuits [9], and screening high secretion cell strains [10]. Among these methods, the uniform water-in-oil microdroplets generated by microfluidic devices have become more popular in recent years because they can better achieve the often-desired single molecule/cell encapsulation [11]. Regarding the required chemicals, fluorinated oils have been more widely used for producing microfluidic microdroplets since 2000 [12,13]. Together with well-designed surfactants, fluorinated oils are more biocompatible and more stable than hydrocarbon oils (e.g., mineral oil, hexadecane), because fluorinated oils are immiscible with either water or hydrocarbons (lipids) [12,14,15].

Many cellular and biochemical assays involve multi-step reactions requiring the addition of certain reagents or chemicals (i.e., Cu^2+^, Mg^2+^ or other metal ions), for example, to start or terminate reactions, lyse cells, facilitate or disrupt protein folding, etc. Microdroplet systems are compatible with reagent delivery during multi-step reactions in principle. On-chip droplet manipulation is currently the main approach to achieve such reagent additions, including picoinjection [9] and droplet fusion [13]. However, it remains challenging to perform these droplet manipulation-based methods, since they usually require specialized microfluidic devices or advanced electric field control, which are not easily accessible to many users. In addition, droplet manipulation-based approaches often have some technical issues, such as low throughput and a time delay of reagent delivery among different microdroplets.

A nanodroplet-based reagent delivery is a simple and promising alternative that does not require any complicated instrumentation. Theoretically, it can enable the simultaneous reagent delivery into entire batches of microdroplets to trigger or inhibit cellular and biochemical assays. Furthermore, this approach can accomplish high-throughput reagent delivery without changing the droplet volume significantly. The nanodroplet-based reagent delivery has so far only been used to add metal ions in water-in-mineral-oil microdroplets generated using bulk methods [4]. It has not been reported whether the delivery of common reagents for multi-step cellular and biochemical reactions such as metal ions or medium-sized biomolecules into water-in-fluorinated-oil microdroplets can be achieved by using nanodroplets.

In this study, we demonstrated the delivery of copper ions and a 20 amino acid-long human p53 peptide into water-in-fluorinated-oil droplets via nanodroplets. We confirmed the delivery of copper ions by microscopic inspection of Cu(OH)_2_ crystal formation on pre-encapsulated iron oxide-containing microbeads under alkaline condition [16]. Furthermore, we confirmed the delivery of the 20 amino acid-long human p53 peptide by detecting fluorescence in water-in-fluorinated-oil microdroplets containing the p53 fluorescent immunosensor [17]. We revealed that the nanodroplet-based reagent delivery was a promising approach for metal ions and medium-sized biomolecules delivery into water-in-fluorinated-oil microdroplets. It has a great potential to be used for performing multi-step cellular and biochemical assays in these kinds of microdroplets such as synchronously triggering the alkyne-azide click reaction [18,19], activation of enzymes [20,21], or the inhibition of enzymatic reactions [22,23].

## 2. Materials and Methods

### 2.1. Materials

Pico-Surf 1 was purchased from Sphere Fluidics (Cambridge, UK). HFE-7500 fluorocarbon oil was purchased from 3M (Maplewood, MN, USA). FluoSurf (2%, *w*/*w*) in HFE-7500 was purchased from Emulseo (Pessac, France). Dynabeads M-270 Epoxy was purchased from Thermo Fisher Scientific (Waltham, MA, USA). Other chemicals used in this study were purchased from Sigma-Aldrich (St. Louis, MO, USA), unless stated otherwise. The 30 μm microfluidic chip was purchased from Dolomite Microfluidics, a brand of Blacktrace Holdings Ltd. (Royston, UK). The 20 μm microfluidic chip (Fluidic 947, Topas) was purchased from microfluidic ChipShop (Jena, Germany).

### 2.2. Preparation of Copper Ion Nanodroplets

To encapsulate the copper ions into nanodroplets, 1 μL of 250 mM CuSO_4_ solution was added into 250 μL HFE-7500 fluorinated oil containing 2% (*w*/*w*) Pico-Surf 1 surfactant. The mixture was then emulsified by vortexing three times at the maximum speed for 1 min with a Vortex-Genie 2 (Scientific Industries, Bohemia, NY, USA). One minute of thorough shaking by hand was performed between vortexing. The resulting clear suspension was used for dynamic light scattering analysis and ion delivery experiments.

### 2.3. Analysis of the Nanodroplet Size with Dynamic Light Scattering

The dynamic light scattering analysis of the sample without dilution was performed on a Microtrac NANO-flex system (Microtrac, York, PA, USA) using a 180° backscatter probe. The refractive index of the dispersed phase was set to 1.33, while the refractive index of the HFE-7500 fluorinated oil was set to 1.29 [24]. The viscosity data were obtained from the product information sheet on the 3M website [25]. The set zero time and run time were 60 s and 90 s, respectively, and the number of runs was two.

### 2.4. Generation of Alkaline Water-in-Fluorinated-Oil Microdroplets and Nanodroplet Delivery

Water-in-fluorinated microdroplets were generated on a 30 μm fluorophilic chip with the μEncapsulator system from Dolomite Microfluidics (Royston, UK). The disperse phase was the alkaline HEPES buffer (20 mM, NaCl 150 mM, pH 9), which was loaded into both channels of the sample reservoir chip. The disperse phase was pre-filtered using a 0.22 μm syringe filter (Foxx Life Sciences, Salem, NH, USA). HFE-7500 fluorinated oil containing 2% (*w*/*w*) Pico-Surf 1 surfactant was used as the continuous phase. Flow rates of disperse phase in both sample channels and continuous phase were set as 2, 2, and 32 μL/min, respectively. The microdroplets were collected in a 1.5 mL centrifuge tube and stored at room temperature in dark until further processing. The microdroplets can be stored for at least a week under the above conditions. To start the reagent delivery, equal volumes of nanodroplets and microdroplets were mixed by gently inverting the tube 5 times. The mixtures were incubated in dark at room temperature. The microdroplets were inspected using a Leica AF 6000 microscope system with the HC PL FLUOTAR 20×/0.50 DRY and HC PL FLUOTAR 40×/0.80 DRY objective lenses (Wetzlar, Germany). The microdroplet size was analyzed with ImageJ [26].

### 2.5. Generation of Immunosensor-Encapsulated Microdroplets and Nanodroplet Delivery

The p53 Quenchbody immunosensor was prepared as described in a previous study [17]. The Quenchbody immunosensor microdroplets were generated on a flow-focusing microfluidic chip with a nozzle size of 20 μm. The disperse phase of 60 nM p53 Quenchbody in PBS buffer (pH 7.4) was loaded into PTFE tubing (inner diameter 0.8 mm) and driven by a syringe pump NE-1000 (New Era, Farmingdale, NY, USA). The HFE-7500 fluorinated oil containing 2% (*w*/*w*) FluoSurf surfactant was used as the continuous phase, which was driven by another syringe pump NE-300 (New Era). Flow rates of disperse phase and continuous phase were set as 1 and 4 μL/min, respectively. The nanodroplet preparation was the same as described above with 424 μM human p53 peptide (EPPLSQETFSDLWKLLPENN) (Lifetein, Hillsborough, NJ, USA) solution in PBS. The delivery procedure was the same as described in the previous section.

The microdroplets were observed using an EVOS FL cell imaging system (Thermo Fisher Scientific) with an RFP light cube using Plan Fluorite 20× objective lens at 100% light intensity. The microdroplet size and fluorescence intensity were analyzed with ImageJ. A circular area with a 35-pixel diameter was used for the fluorescence intensity calculation, and the integrated intensity in the red channel was analyzed from three microscopic views of three independent cell counting chambers (Disposable Hemocytometer, Funakoshi, Tokyo, Japan). According to the fluorescence intensity of the droplets in the maximum-response sample, the droplets with an intensity >10,000 a.u. were identified as spiked maximum-response ones in the mixture samples of maximum-response and sensor droplets.

### 2.6. Measurement of Dose–Response Curve of p53 Quenchbody

The p53 Quenchbody solution (final concentration, 60 nM) was mixed with different concentrations of human p53 peptide in PBS at 25 °C. The fluorescence intensity of a 60 μL reaction was measured using a CLARIOstar microplate reader (BMG Labtech, Ortenberg, Germany) with excitation and emission wavelengths of 535/20 nm and 585/30 nm (center/bandwidth), respectively. The peptide concentration and the normalized fluorescence intensity were fitted to a four-parameter logistic Equation (1) using ImageJ. The parameters a, b, c, and d correspond to the minimum value, hill slope, EC50, and maximum value, respectively.

(1) $$y\;=\;d+\frac{a-d}{1+{\left(\frac{x}{c}\right)}^{b}}$$

## 3. Results and Discussion

### 3.1. Preparation and Characterization of Microdroplets and Copper Ion Nanodroplets

**Figure 1:**
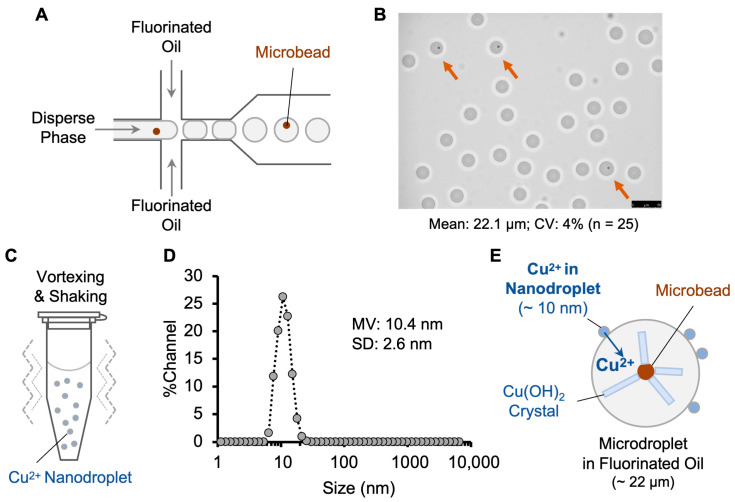
Copper ion delivery into water-in-fluorinated-oil droplets via nanodroplets. (**A**) Generation of uniform water-in-fluorinated-oil microdroplets by flow-focusing microfluidic device. (**B**) Microscopy image of microdroplets. Orange arrows indicate the microdroplets containing a microbead. Scale bar, 25 μm. CV: coefficient of variation. (**C**) Preparation of copper ion nanodroplets by vortexing. (**D**) Size distribution of the copper ion nanodroplets analyzed by dynamic light scattering. MV: mean volume diameter. SD: standard deviation. (**E**) Delivery of copper ions into microdroplets through co-incubation leading to crystal formation on microbeads, thereby confirming successful delivery to copper ions.

The water-in-fluorinated-oil microdroplets were prepared by the flow-focusing method [9] on a 30 µm microfluidic chip (Figure 1A). The dispersed and continuous phases were microbead-suspended alkaline buffer and HFE-7500 fluorinated oil with 2% (*w*/*w*) Pico-Surf 1 surfactant, respectively. The average diameter of the microdroplets was 22.1 μm, with a 4% coefficient of variation (CV) (Figure 1B).

The copper ion nanodroplets were prepared by emulsifying 1 µL of copper sulfate solution in 250 µL HFE-7500 fluorinated oil containing the same surfactant as above (Figure 1C). The size of the nanodroplets was determined by dynamic light scattering (DLS) (Figure 1D). The nanodroplets had a mean volume diameter of 10.4 ± 2.6 nm, which falls in the common range of 10–200 nm reported in previous studies [18,19]. Nanodroplet size can be influenced by several factors, including relative viscosity, surfactant concentration, and vortex time [20,22]. Low-energy methods such as vortexing and manual shaking usually generate smaller nanodroplets compared to high-energy methods of sonication and high-pressure homogenization [20].

### 3.2. Copper Ion Delivery and Confirmation with Crystal Formation

**Figure 2:**
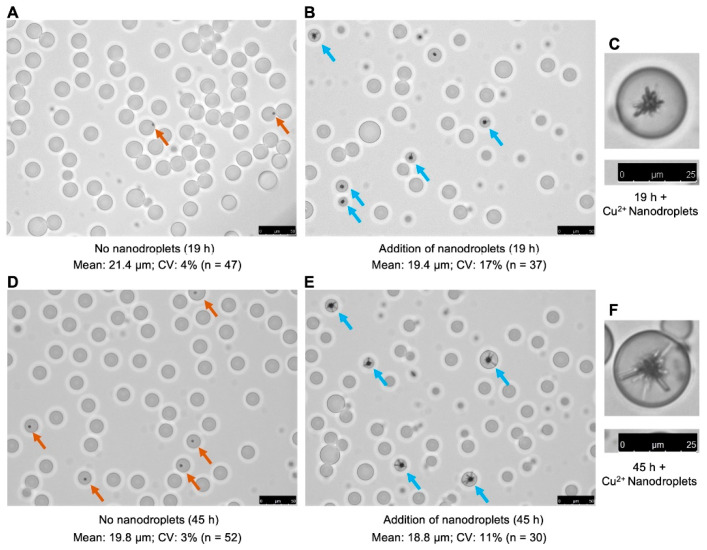
Confirmation of the copper ion delivery into water-in-fluorinated-oil microdroplets via crystal growth on microbeads. (**A**) Incubation for 19 h in the absence of copper nanodroplets, 20× objective lens. (**B**) Incubation for 19 h in the presence of copper nanodroplets, 20× objective lens. (**C**) Cropped single microdroplet image after 19 h incubation in the presence of copper nanodroplets, 40× objective lens. (**D**) Incubation for 45 h in the absence of copper nanodroplets, 20× objective lens. (**E**) Incubation for 45 h in the presence of copper nanodroplets, 20× objective lens. (**F**) Cropped single microdroplet image after 45 h incubation in the presence of copper nanodroplets, 40× objective lens. Orange arrows indicate the microdroplets containing microbeads under the control condition (without nanodroplets). Blue arrows indicate the droplets showing crystal formed on microbeads. Scale bar for 20× objective lens, 50 μm; scale bar for 40× objective lens, 25 μm.

The copper ion delivery (Figure 1E) was achieved by simply mixing the nanodroplet and microdroplet solutions at a 1:1 volume ratio. The emulsion mixture was gently inverted 5 times and incubated in the dark as recommended by the manufacturer of the surfactant and oil. The emulsion mixture was analyzed with a microscope after 19 h (Figure 2A–C) and 45 h of incubation (Figure 2D–F). The formation of clear crystals on the microbeads was observed after 19 h of incubation with copper ion nanodroplets, which proved the successful delivery of copper ions into the water-in-fluorinated-oil droplets. The crystals had grown in size by 45 h of incubation. However, a size change in the microdroplets was also observed after incubation with nanodroplets for both 19 and 45 h, which could be due to crystal- and nanodroplet-mediated droplet coalescence. Overall, we demonstrated the delivery of metal ions into water-in-fluorinated-oil microdroplets using nanodroplets. Furthermore, the label-free approach we used to confirm the copper ion delivery into microdroplets could potentially be adapted and used in combination with other metal ions and crystal formation conditions.

### 3.3. Peptide Delivery and Confirmation Using a Fluorescent Immunosensor

**Figure 3:**
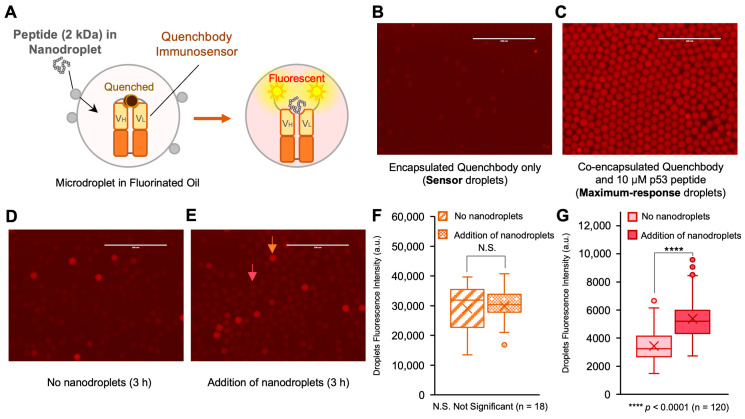
Nanodroplet-based peptide delivery into water-in-fluorinated-oil microdroplets. (**A**) Scheme of nanodroplet-based peptide delivery and visualization by immunosensor (Quenchbody). VH and VL, variable region of heavy chain and light chain of antibody. Without peptide, the fluorescence is weak due to the dye–dye quenching and photoinduced electron transfer from tryptophan residues in antibody fragments. The presence of peptide separates fluorophores, leading to a fluorescent signal. (**B**) Microdroplets containing Quenchbody only (sensor droplets). (**C**) Microdroplets containing both Quenchbody and 10 μM human p53 peptide (maximum-response droplets; positive control). The maximum-response droplets are spiked into the sensor droplets as internal control during fluorescence imaging. (**D**) Incubation of mixed microdroplets (90% sensor droplets and 10% maximum-response droplets) in absence of nanodroplets after 3 h. All three microscopic views for this analysis are shown in Figure S1A. (**E**) Incubation of mixed microdroplets with p53 peptide-containing nanodroplets after 3 h. The orange arrow indicates one of the positive control droplets. The pink arrow indicates one of the sensor droplets after peptide delivery. All three microscopic views for this analysis are shown in Figure S1B. Scale bar, 200 μm. (**F**) Box plot of fluorescence intensity of the maximum-response droplets after 3 h incubation. (**G**) Box plot of fluorescence intensity of sensor droplets after 3 h incubation. Box plots indicate the median (center line), mean (cross), first and third quartiles (box edges) and full data ranges (whiskers), and outlier (circles). The level of significance was determined by two-tailed Welch’s *t*-test.

We performed a similar protocol to verify the delivery of a 20-amino-acid-long human p53 peptide into water-in-fluorinated-oil microdroplets using nanodroplets (Figure 3A). The aqueous phase containing the human p53 protein fluorescent immunosensor (p53 Quenchbody) [17] was used to prepare the microdroplets using the same flow-focusing method as described for the copper ion delivery above. We prepared two kinds of microdroplets: sensor microdroplets to detect peptide delivery and maximum response microdroplets as positive control. The sensor microdroplets were defined as the ones containing the p53 Quenchbody but without p53 peptide, which had only weak fluorescence intensity (Figure 3B). However, when the p53 Quenchbody binds to the human p53 peptide, the complex shows strong fluorescence. The maximum response microdroplets (positive control) were prepared by encapsulating the Quenchbody together with the p53 peptide. Due to the high concentration of the human p53 peptide encapsulated in positive control microdroplets, these droplets showed a >10-fold higher fluorescence intensity compared to the sensor microdroplets (Figure 3C). The mean diameter of the microdroplets was 21.5 μm (CV 6%). Before adding nanodroplets to the microdroplets for the peptide delivery, positive control microdroplets were mixed with sensor microdroplets in a ratio of 1:9, which served as internal controls for fluorescence intensity comparison among different samples, and to facilitate microscopic image analysis. The microdroplet mixture was incubated with or without nanodroplets in the dark for 3 h to evaluate the peptide delivery. The microscopy images of both samples are shown in Figure 3D and Figure 3E, respectively. The positive control microdroplets (e.g., the droplet marked with orange arrow in Figure 3E) showed bright red fluorescence (intensity > 10,000 a.u.), which did not significantly change after the addition of nanodroplets (Figure 3F). However, the fluorescence intensity of the sensor droplets (e.g., the droplet marked with pink arrow in Figure 3E) increased by 1.6-fold, thereby suggesting the successful peptide delivery (Figure 3G). The average peptide concentration inside the microdroplets after 3 h was ~28 nM, as determined by comparison to a standard curve measured in bulk condition (Figure S2). The peptide concentration increased only slightly to 34 nM when the incubation was continued for a total of 24 h (Figure S3), which suggested that the peptide delivery was mostly completed in the first 3 h. These results demonstrated the feasibility of using nanodroplets to achieve delivery of medium-sized biomolecules into water-in-fluorinated-oil microdroplets.

Recent reports have described a sodium dodecyl sulfate (SDS)-triggered cargo release approach for the synchronized reagent delivery in microdroplets to facilitate multi-step bioassays in a water-in-oil-in-water double emulsion (DEs) system [27]. In the reported approach, liposomes containing the reaction reagents were co-encapsulated in the aqueous core of DEs. The addition of SDS to the outer aqueous medium resulted in some of the SDS diffusing into the inner aqueous core of DEs to lyse the liposomes. The lysis caused the release of the liposome’s contents, thereby triggering the corresponding bioassays at a desired time point. However, this method is only suitable for relatively hydrophilic or large molecules, but is not compatible with compounds that are phospholipid membrane-permeable due to the properties of liposomes. Furthermore, the biochemistry assay in the DEs must be compatible with the ionic surfactant SDS. The nanodroplet-based reagent delivery method described in our current study is a straightforward alternative to achieve multi-step bioassays, yet without introducing the additional trigger molecules (e.g., SDS) in water-in-oil microdroplets or a two-step DE generation system [9]. In the future, the systematic evaluation of the effects of surfactant concentration, size of the droplet, and molecular weight of the reagents to be delivered could be performed with the methods provided in this study to gain a deeper understanding of the mechanism and limitation of the nanodroplet-based reagent delivery.

Semi-permeable hydrogels [28] or microcapsules [29] are another state-of-the-art approach for controlling the biochemical reactions within micro-compartments. In these systems, the different reagents smaller than the pore size can diffuse into micro-compartments. Different from the reagent delivery demonstrated in this study, the semi-permeable micro-compartments allow molecule exchange by changing the suspension buffers. In the meantime, the products or targeting compounds must be larger than the pore size to be retained in the semi-permeable hydrogels or microcapsules, which sometimes limits their applications.

During the recent COVID-19 pandemic, a one-pot biochemical assay for nucleic acid detection using loop-mediated isothermal amplification (LAMP) and CRISPR-based sensing system was developed [30,31]. This type of system utilized Mg^2+^-dependent Cas enzymes to give a fluorescent signal after recognizing the LAMP-amplified target nucleic acid sequences. These methods can be used for digital nucleic acid detection after being encapsulated into microdroplets, but the more precise control of the Cas activity will be preferred to reduce the background by triggering the enzyme activity after the microdroplet generation. This could potentially be achieved by using the approach presented in this study to deliver the Mg^2+^ ions into the sensor droplets.

## 4. Conclusions

We demonstrated that nanodroplets can be used as carriers for metal ions and delivery of medium-sized biomolecules (2 kDa peptide) into water-in-fluorinated-oil microdroplets. This facile nanodroplet preparation and delivery procedure is easy to access for many researchers, because it does not require specialized equipment or a complicated setup. We believe that this nanodroplet-based delivery technique is a promising approach capable of achieving multi-step cellular and biochemical assays in artificial reaction compartments for a broad range of biological applications, including molecular evolution, cell factory engineering, digital nucleic acid detection, or drug screening.

## Data Availability

The data presented in this study are available in the article or Supplementary Material.

## Supplementary Materials

The following supporting information can be downloaded at: https://www.mdpi.com/article/10.3390/bios13080768/s1, Figure S1: Confirmation of the p53 peptide delivery with p53 Quenchbody in droplets; Figure S2: Dose-dependency of p53 Quenchbody immunosensor in PBS buffer; Figure S3: Nanodroplet-based peptide delivery into water-in-fluorocarbon microdroplets after 24 h incubation.
