# Supplementary material for: Nanodroplet-Based Reagent Delivery into Water-in-Fluorinated-Oil Droplets
Source: Biosensors (Basel). 2023 Jul 28;13(8):768. doi: 10.3390/bios13080768 (PMC10452409; doi:10.3390/bios13080768)
Supplement: Supplementary file 1 [file biosensors-13-00768-s001.zip › biosensors-2461289-SI.pdf]

Supplementary Materials for

Nanodroplet-based reagent delivery into water-in-fluorinated-oil droplets

Bo Zhu [a, b, c, *, †], Zhe Du [b, d, †], Yancen Dai [c], Tetsuya Kitaguchi [c], Sebastian Behrens [b, e] and Burckhard Seelig [a, b, *]

[a] Department of Biochemistry, Molecular Biology and Biophysics, University of Minnesota, Minneapolis, MN 55455, USA.

[b] BioTechnology Institute, University of Minnesota, St. Paul, MN 55108, USA.

[c] Laboratory for Chemistry and Life Science, Institute of Innovative Research, Tokyo Institute of Technology, Yokohama 226-8503, Japan.

[d] Center for Environmental Health Risk Assessment and Research, Chinese Research Academy of Environmental Sciences, Beijing 100012, China.

[e] Department of Civil, Environmental, and Geo- Engineering, University of Minnesota, Minneapolis, MN 55455, USA.

† These authors contributed equally to this work.

*** Correspondence:**

Bo Zhu: zhu.b.ac@m.titech.ac.jp

Burckhard Seelig: seelig@umn.edu

Supplementary Figures

A

B

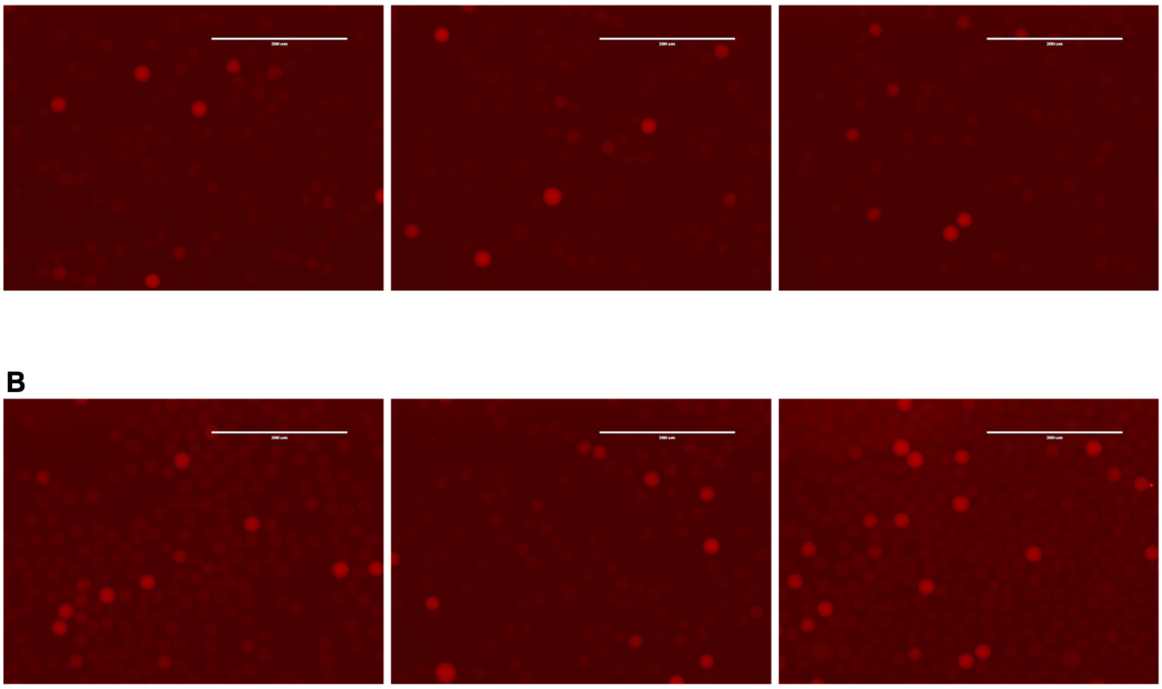

Figure S1. Confirmation of the p53 peptide delivery with p53 Quenchbody in droplets. Incubation of mixed microdroplets (90% sensor droplets and 10% maximum-response droplets) in absence of peptide nanodroplets (A) or in the presence of peptide nanodroplet (B) after 3 h. Scale bar, 200 µm.

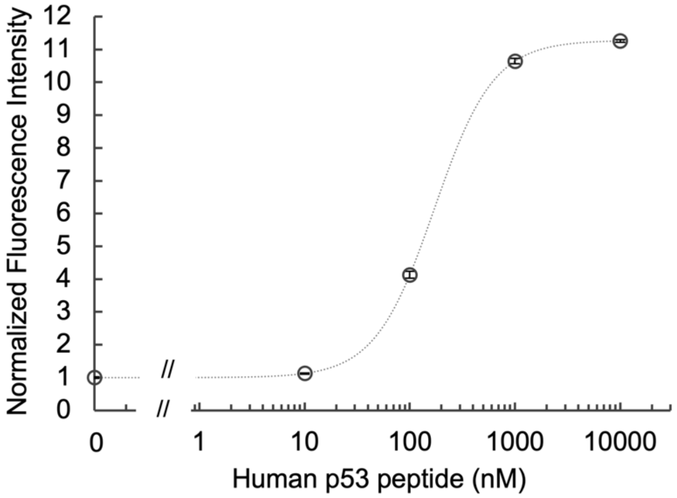

Figure S2. Dose-dependency of p53 Quenchbody immunosensor in PBS buffer. Quenchbody concentration: 60 nM; n = 2; Open circle, data points. Dot line, fitting curve, Minimum: 1.00, Maximum: 11.3, EC50: 171 nM, hill slope: 1.54. Data are shown as mean ± standard deviation.

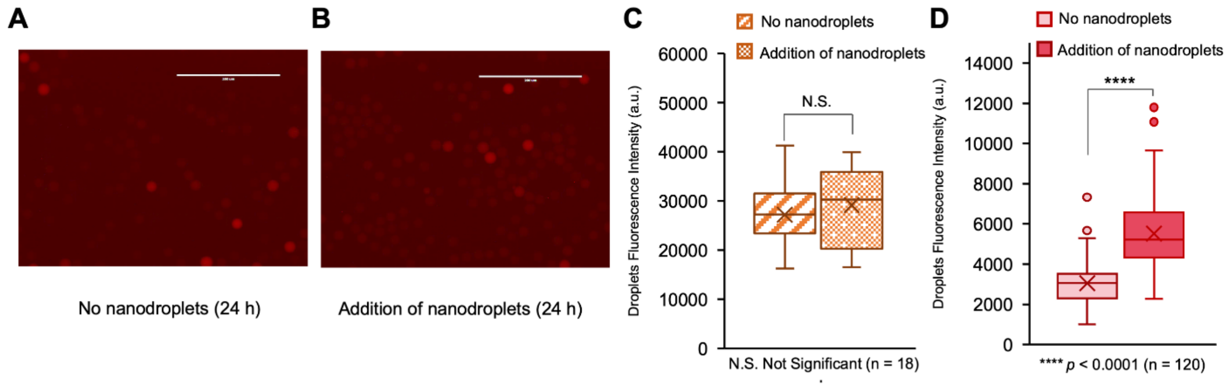

Figure S3. Nanodroplet-based peptide delivery into water-in-fluorocarbon microdroplets after 24 h incubation. The maximum-response droplets (microdroplets co-encapsulated with sensor and 10 µM p53 peptide) are spiked into the sensor droplets (microdroplets with encapsulated sensor only) as internal control during fluorescence imaging. (A) Incubation of mixed microdroplets (90% sensor droplets and 10% maximum-response droplets) in the absence of nanodroplets after 24 h. (B) Incubation of mixed microdroplets with p53 peptide-containing nanodroplets after 24 h. Scale bar, 200 µm. (C) Box plot of fluorescence intensity of the maximum-response droplets after 24 h incubation. (D) Box plot of fluorescence intensity of sensor droplets after 24 h incubation. Box plots indicate the median (centre line), mean (cross), first and third quartiles (box edges) and full data ranges (whiskers), and outlier (circles). The level of significance was determined by two-tailed Welch's *t*-test.
